# CdSe/ZnS Quantum Rods (QRs) and Phenyl Boronic Acid BODIPY as Efficient Förster Resonance Energy Transfer (FRET) Donor–Acceptor Pair

**DOI:** 10.3390/nano14090794

**Published:** 2024-05-03

**Authors:** Gianluca Salerno, Pasquale Palladino, Marcello Marelli, Laura Polito, Maria Minunni, Debora Berti, Simona Scarano, Giacomo Biagiotti, Barbara Richichi

**Affiliations:** 1 Department of Chemistry “Ugo Schiff”, University of Firenze, Via della Lastruccia 13, Sesto Fiorentino, 50019 Firenze, Italy; 2 Istituto di Scienze e Tecnologie Chimiche “Giulio Natta” del Consiglio Nazionale delle Ricerche (SCITEC-CNR), Via G. Fantoli 16/15, 20138 Milan, Italy; 3 Department of Pharmacy, University of Pisa, Via Bonanno 6, 56126 Pisa, Italy

**Keywords:** PBA-BODIPY, quantum dots, quantum rods, FRET, responsive nanomaterial, fluorescence

## Abstract

The reversibility of the covalent interaction between boronic acids and 1,2- or 1,3-diols has put the spotlight on this reaction for its potential in the development of sensors and for the fishing of bioactive glycoconjugates. In this work, we describe the investigation of this reaction for the reversible functionalization of the surface of CdSe/ZnS Quantum Rods (QRs). With this in mind, we have designed a turn-off Förster resonance energy transfer (FRET) system that ensures monitoring the extent of the reaction between the phenyl boronic residue at the *meso* position of a BODIPY probe and the solvent-exposed 1,2-diols on QRs’ surface. The reversibility of the corresponding boronate ester under oxidant conditions has also been assessed, thus envisioning the potential sensing ability of this system.

## 1. Introduction

The reversible covalent interaction of boronic acids (BA) with 1,2- and 1,3-diols has been exploited in many different research areas, and it has facilitated the development of sensors, separation techniques, and lately of antibody–drug conjugates and biomaterials [1,2]. BA-diols binding is regulated by the pH, and the resulting five- or six-membered cyclic boronate esters show a boron atom with a sp^3^ hybridization and an increased acidity compared to the parent boronic acid [3,4,5]. The dynamic equilibria that regulate the formation of boronate esters have also been widely investigated in the reaction with carbohydrates. This approach has opened a vibrant and new area of scientific research focused on the discovery of sensors for saccharides and saccharide-containing molecules, ranging from the most investigated sensors for glucose to the latest for sialic acid and sialic acid-bearing glycoconjugates [3,6,7,8]. Moreover, boronic acid-based matrices for the purification of glycoproteins appeared on the market, and boronic acid-based tools for the fishing of glycoproteins [9,10,11], the labeling of specific carbohydrate epitopes [10], and fluorescent biomaterials for drug delivery have been developed [12]. Furthermore, the reactions of boronic and phenyl-boronic (PBA) acids with catechols have received much attention in the sensing of catecholamines [13,14], and for the preparation of fluorescent and stimuli-responsive nanomaterials [15,16,17]. Therefore, the investigation of the reactivity of boronic acid with different matrices is currently of significant interest [18,19], and it can open new perspectives and applications for such versatile tools.

**Figure 1:**
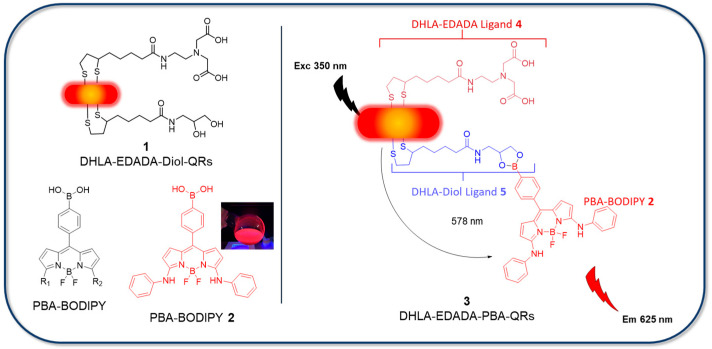
Schematic representation of CdSe/ZnS QRs **1,** general structure of our PBA-BODIPY [20], PBA-BODIPY **2** [20], and the QRs-based FRET system **3**.

In this context, we recently reported the straightforward synthesis of a family of modular and functional PBA-BODIPY dyes (general structure, Figure 1) [20].

We demonstrated that the emission bands of the PBA-BODIPYs can be easily tuned according to the substitution pattern at the 3,5 positions of the BODIPY core [20]. Moreover, the reactivity of the PBA moiety vs. 1,2-diols was widely investigated in different settings, and the formation of the boronate esters with the glycan chains of an antibody [20], the hydroxyl groups of the dextran polysaccharide [12], and the hydroxyl/phenol groups of the surface of graphene oxide [15,16,20,21] was demonstrated using conventional and mechanochemical approaches. 

In line with this, we report here on the investigation of the receptor-like ability of the PBA moiety with solvent-exposed 1,2-diols on the core/shell CdSe/ZnS Quantum Rods (QRs) surface (compound **1**, Figure 1). The surface functionalization of the QRs and thus the formation of the boronate esters was monitored by the proper design of a turn-off Förster resonance energy transfer (FRET) system [22,23]. In general, for the FRET process, a donor is excited at a defined wavelength, and the energy is transferred, via non-radiative dipole–dipole interactions, to a properly selected acceptor, which emits through radiative relaxation. This process efficiency is directly related to the spectral overlapping of donor emission and acceptor absorbance. Donors with high quantum yields maximize the process efficiency [22,23]. Indeed, QDs and QRs have been widely engaged as excellent FRET donors, and the Förster transfer mechanism has been mainly investigated for the development of quantum dots (QDs)–organic dyes for FRET-based biosensors [24]. 

With this in mind, in this work, PBA-BODIPY **2** was selected to be coupled to CdSe/ZnS QRs **1** (Figure 1) for setting up a perfect FRET pair, and the Forster transfer mechanism was used to monitor the extent of functionalization. Indeed, QDs and QRs, compared to organic molecule donors, minimize the acceptor direct excitation thanks to the broad absorption, i.e., they are excited in a large part of the UV–vis spectrum. On the other hand, the PBA-BODIPY structural modularity allows fine-tuning of the absorbance spectrum to the FRET acceptor by targeted substitution. Accordingly, PBA-BODIPY **2** and CdSe/ZnS QRs **1** have been synthesized and further used to prepare the PBA-BODIPY-QRs conjugate **3,** which proved to successfully work as an acceptor–donor FRET pair.

## 2. Materials and Methods

All reagents were purchased from Sigma-Aldrich (St. Louis, MO, USA), and they have been used without any further purification, if not specified otherwise. UV–vis spectra were recorded on a Varian Cary 4000 UV–vis spectrophotometer (Varian, Palo Alto, CA, USA) using a 1.0 cm cell. Fluorescence spectra were registered on a Jasco FP750 spectrofluorometer (Jasco, Easton, MD, USA) using a 1.0 cm cell. NMR spectra were recorded on Varian Inova 400 and/or Varian Mercury plus 400 instruments. Chemical shifts were reported in parts per million (ppm) relative to the residual solvent peak, rounded to the nearest 0.01 for proton and 0.1 for carbon reference: CHCl_3_ [^1^H: 7.26 ppm, ^13^C: 77.0 ppm]. Coupling constants J were reported in Hz to the nearest 0.01 Hz. Peak multiplicity was indicated as follows: s (singlet), d (doublet), t (triplet), q (quartet), m (multiplet), and br (broad signal). ESI-MS was recorded on the LC-MS LCQ Fleet ThermoFisher Scientific (Waltham, MA, USA). Centrifugations were performed on an AFI SIRENA centrifuge (AFI, Château-Gontier-sur-Mayenne, FR).

### 2.1. Dynamic Light Scattering

Dynamic light scattering of QRs in water was employed to determine the apparent hydrodynamic size of the QRs and to check for particle aggregation. Measurements were carried out on a Brookhaven 90Plus (BrookHaven, Holtsville, NY, USA) particle sizer equipped with a red laser (659 nm) and an APD detector placed at 90°. Samples were enclosed in a PMMA cell and placed in a temperature-controlled (25 °C) measurement camera. The measured autocorrelation functions were fitted with the non-negatively constrained least squares algorithm (NNLS) to extract the diffusion coefficients of the particles as an average of 5 independent measurements. Diffusion coefficients were then converted into apparent hydrodynamic size through the Stokes–Einstein law. Zeta-potentials of QRs in an aqueous solution were obtained via phase analysis light scattering (PALS) using a Brookhaven ZetaPALS instrument. The apparatus consisted of a laser operating at 659 nm and an APD detector placed at 15°. The recorded electrophoretic mobilities (as an average of 5 independent measurements) were converted into ζ-potential values through the Helmholtz–Smoluchowski equation.

### 2.2. Transmission Electron Microscopy (TEM) and Scanning Electron Microscopy (STEM)

TEM and STEM micrographs were acquired using a ZEISS LIBRA200FE (Carl Zeiss AG, Oberkochen, DE) operating at 100 kV. The specimens were prepared by dropping a dilute suspension of QRs (50 µg/mL) onto a 200-mesh carbon-coated copper grid and drying it overnight before performing the analysis. The nanoparticle size measurements on the micrographs were performed using Olympus SIS iTEM software version 5.1.

### 2.3. UV–Visible and Fluorescence Emission Spectroscopy

QRs were dissolved in the desired solvent at 0.1 mg/mL or 0.05 mg/mL concentration using an ultrasound bath (59 kHz, 5 min continuous mode), and absorption or emission spectra were recorded.

#### 2.3.1. Synthesis of **5**

To a stirred solution of dihydrolipoic acid DHLA (250 mg, 1.21 mmol) in dry dimethylformamide (3 mL), 1,1′-carbonyl diimidazole (30 mg, 1.82 mmol) and 4-methyl morpholine (180 mg, 1.82 mmol) were added. The reaction mixture was stirred at r.t. for 30’, then it was dropwise added to a solution of 3-amino-1,2-propanediol **6** (140 mg, 1.57 mmol) in dry dimethylformamide (0.8 mL). The reaction mixture was stirred at r.t. for 3 h, diluted with ethyl acetate (100 mL), and washed with water (5 × 10 mL) and brine (2 × 10 mL). The organic phase was dried over sodium sulfate, filtered, and reduced in vacuum to give **5** (205 mg, 60% yield) as brown oil that was used in the next reaction without further purification. ^1^H NMR (400 MHz, CD_3_OD) δ: 3.73–3.64 (m, 1H, H-9), 3.62–3.53 (m, 1H, H-3), 3.53–3.43 (m, 2H, H-8), 3.34 (dd, *J* = 13.8 Hz, 4.9 Hz, 1H, H-10_a_), 3.25–3.03 (m, 3H, H-1, and H-10_b_), 2.51–2.41 (m, 1H, H-2_a_), 2.22 (t, *J* = 7.4 Hz, 2H, H-7), 1.94–1.83 (m, 1H, H-2_b_), 1.79–1.55 (m, 4H, H-4, and H-6), 1.53–1.38 (m, 2H, H-5). ^13^C NMR (101 MHz, CD_3_OD) δ: 175.19, 70.64, 63.61, 56.11, 41.93, 39.87, 37.92, 35.37, 34.32, 28.47, 25.28. ESI-MS *m*/*z*: calcd. for C_11_H_22_NO_3_S_2_+ [M + H^+^]^+^ 280.10 found 280.13.

#### 2.3.2. Synthesis of CdSe/ZnS QRs **7**

CdSe/ZnS QRs were prepared according to the literature with minor changes [25,26,27,28]. Tributylphosphine (TBP) (5 mL) was added into a vessel containing elemental selenium (Se) (39.5 mg, 0.50 mmol) under argon to give a 0.1 M Se solution. Cadmium oxide (37 mg, 0.30 mmol), tetradecylphosphonic acid (TDPA) (334 mg, 1.2 mmol), and trioctylphosphine oxide (TOPO) (3.6g, 9.3 mmol) were dried in vacuum at 40 °C for 15 min and then at 60 °C for 15 min. The atmosphere was exchanged for Ar, and the solution was heated to 300 °C. When the solution turned colorless, the Se solution (2.5 mL, 0.25 mmol) was added. After 3 min, the reaction was quenched by rapid cooling. Particles were precipitated by the addition of MeOH (10 mL), centrifuged (4 min, 6000 rpm), and the supernatant discarded. The particles were then redissolved in CHCl_3_ (10 mL). CdSe/ZnS QRs were prepared according to the literature with minor changes [29]. CdSe QRs solution (8 mL, 0.2 mmol) was added to TOPO (3.6 g, 1 mmol), and the CHCl_3_ evaporated under vacuum at 50 °C. The resultant material was then heated to 180 °C under Ar. In a separate flask, to a degassed (Ar atmosphere for 30 min) solution of zinc stearate (198 mg, 0.313 mmol) in toluene (5 mL), was added hexamethydisilathiane (0.05 mL, 0.2 mmol). The resulting ZnS solution (0.7 mL, 0.03 mmol) was added dropwise to the heated CdSe QRs solution and mixed for 2 h at 100 °C. The mixture was then allowed to cool to room temperature, and the particles precipitated with the addition of MeOH (10 mL). The mixture was centrifuged (4 min, 6000 rpm), and the supernatant was discarded before redissolving the CdSe/ZnS QRs **7** in CHCl_3_.

#### 2.3.3. Synthesis of QRs **1**

QRs **1** were prepared according to a previously reported protocol with minor revisions [29]. To a stirred solution of **4** (150 mg, 0.41 mmol) and **5** (114 mg, 0.41 mmol) in a mixture of MeOH/H_2_O (1/1, *v*/*v*, 3 mL), NaBH_4_ (93 mg, 2.5 mmol) was added under a nitrogen atmosphere, and the mixture was stirred for 1.5 h. The pH of the solution was then adjusted to neutral by the dropwise addition of HCl (1 M in H_2_O) before transferring it to a glass vial. Moreover, 1 mL of CdSe/ZnS QRs **7** dispersion in chloroform (10 mg/mL) was added to the vial, and the biphasic mixture was stirred vigorously for 17 h. Once the colored QRs moved to the aqueous layer, the chloroform phase was removed, and the aqueous layer was dialyzed for 16 h and then freeze-dried to obtain 12 mg of QRs **1** as a red lyophilized powder. UV–vis absorption λ_max_ = 561 nm, fluorescence emission λ = 578 nm. DLS: 38.4 ± 3.2 nm. ζ-potential: −18 ± 2 mV.

#### 2.3.4. Synthesis of QRs **3**

To a stirred suspension of **1** (10 mg) in H_2_O (1 mL), a solution of **2** (0.9 mg, 0.001 mmol) in MeOH (0.5 mL) was added, and the reaction mixture was stirred at r.t. for 24 h. Then, the reaction mixture was centrifuged at 5000 rpm for 20 min, and the supernatant was removed. The solid was then dispersed in methanol (3 mL) and centrifuged. Finally, the solid was dispersed in 3 mL of water, dialyzed for 24 h, and freeze-dried to obtain 9 mg of blue lyophilized powder. Fluorescence emission λ = 625 nm. DLS: 38.4 ± 3.2 nm. ζ-potential: −39 ± 4 mV.

## 3. Results and Discussion

**Scheme 1:**
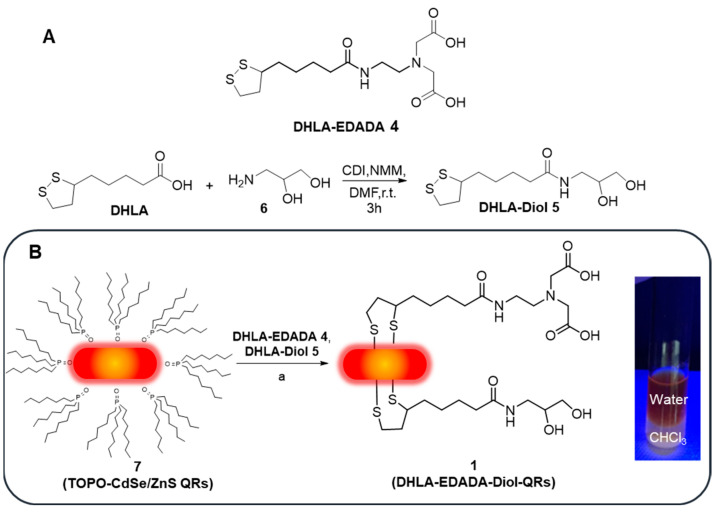
(**A**) Structures of the DHLA-EDADA **4** and synthesis of the DHLA-diol **5** ligand; (**B**) Ligand exchange reaction for the preparation of DHLA-EDADA-diol-QRs **1**. Reaction conditions. (**A**) **4**:**5** molar ratio 1:1, methanol/water 1:1, NaBH_4_, r.t. for 17 h.

The water-soluble CdSe/ZnS QRs **1** bearing a binary mixture of two thiol-containing heterobifunctional coating ligands **4**–**5** were prepared (Scheme 1A). Ligands **4**–**5** contain a common dihydrolipoic acid (DHLA) molecule that provides thiol groups with a high affinity for the nanocrystal surface. Then, the DHLA was conjugated to an ethylenediamine-N,N-diacetic acid residue (EDADA), affording the DHLA-EDADA ligand **4** that ensures the colloidal stability of the QR dispersions [29]. The specific length of the ligand for the conjugation of PBA-BODIPY **2** was chosen according to the FRET theory, keeping the fluorophore donor and a fluorophore acceptor in close proximity (not farther than 10 Å), ensuring a good FRET effect [24,30]. Accordingly, the DHLA-EDADA **4** was combined in a binary mixture with the DHLA-diol ligand **5** (Scheme 1A), bearing the terminal 1,2-diol groups. 

The DHLA-EDADA ligand **4** was prepared according to our previously published protocol [29], whereas the DHLA-diol ligand **5** was synthesized by reacting the DHLA with the 3-amino-1,2-propanediol **6** using carbonyl diimidazole (CDI) to activate the carboxylic group of the DHLA (Scheme 1A).

**Figure 2:**
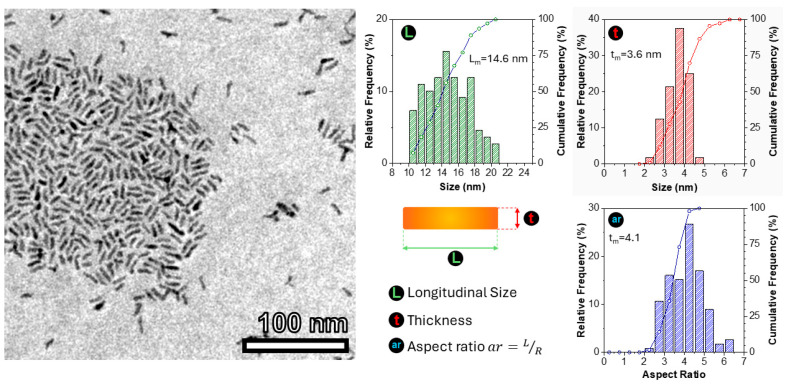
TEM (**left side**) micrographs of QRs **7** and related size distribution (**right side**).

Trioctylphosphine Oxide (TOPO)-coated CdSe/ZnS QRs **7** were synthesized using the hot-injection method previously reported [29]. According to the literature [31,32], electron microscopy analysis highlights an elongated rod-like morphology for the CdSe/ZnS QRs **7**. QRs appear quite homogeneous in size and shape (Figure S1, see Supplementary Materials), and TEM measurements point out an estimated longitudinal mean size of 14.6 nm, a mean thickness of 3.6 nm, and a mean aspect ratio of 4.1 (Figure 2).

The QRs showed a narrow size range in thickness (from 2 to 5 nm), while the longitudinal size span was in a range from 10 to 20 nm, where almost 80% of the nanoparticles had a length below 17 nm. Then, a binary mixture of the ligands **4**:**5** (1/1 ratio) was used for the ligand exchange step with TOPO-coated core/shell CdSe/ZnS quantum dots **7** [28] (Scheme 1B). The reductive opening of the disulfide bond of the DHLA residues of both ligands **4** and **5** with sodium borohydride (see experimental) afforded the bidentate thiol groups, which can stably bind the zinc-coated QRs, replacing the hydrophobic TOPO ligand. The ligand exchange was easily followed by the shift of the fluorescent nanoparticles from the organic to the water phase (Scheme 1B). The resulting QRs **1** were purified by dialysis (see experimental) and freeze-dried to afford the water-soluble fluorescent nanoparticles as a red powder.

**Figure 3:**
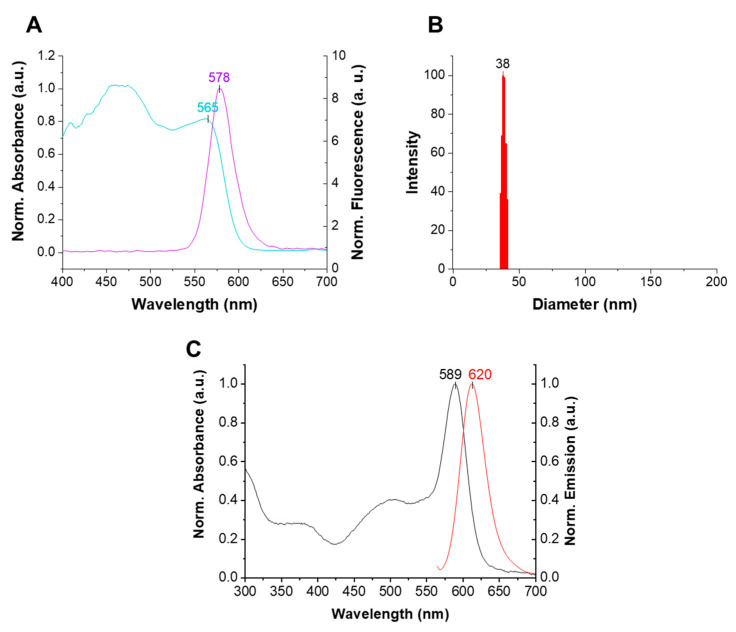
Properties of QRs **1** dispersion in water (0.05 mg/mL): (**A**) UV–vis absorption (cyan) and fluorescence emission spectra (purple) (excitation λ_exc_ = 350 nm; λ_em_ = 578 nm). (**B**) apparent hydrodynamic size of QRs **1** measured by DLS analysis. (**C**) UV–vis absorption spectrum (black) and fluorescence emission spectrum (red) of PBA-BODIPY **2** (5 µM in methanol) [20].

As expected, QRs **1** showed a broad absorption spectrum in the UV–vis region and a narrow emission with a maximum at λ = 578 nm (Figure 3A). The nanoparticles’ size was estimated by dynamic light scattering (DLS), an analysis measuring the hydrodynamic radius that was found to be 38.4 nm (Figure 3B, Figure S2, see Supplementary Materials). As usual, the primary rod-like particles display a larger apparent hydrodynamic size with respect to the size measured by TEM, consistent with some polydispersity, the added hydrodynamic thickness of the ligand shell, and possibly the minor presence of some aggregates. The zeta (ζ) potential measurements showed a negative (−18 ± 2 mV) surface charge due to the presence of carboxylic groups in the DHLA-EDADA ligand **4**.

The QRs **1** surface coating was also confirmed by ^1^H-NMR investigation (see Supplementary Materials, Figures S3 and S4), and the 1:1 ratio of the **4**:**5** mixture was confirmed by the integrals of the ^1^H-NMR signals at 4.00–3.94 ppm, assigned to the C*H*-OH of DHLA-diol **5**, and bs at 2.17 ppm, assigned to C*H*_2_ α to the carbonyl group of the dihydrolipoic acid. The integral ratio between these two NMR peaks indicates a controlled functionalization of the nanoparticles’ surface with a molar ratio between ligands **4** and **5** of 1:1.

**Figure 4:**
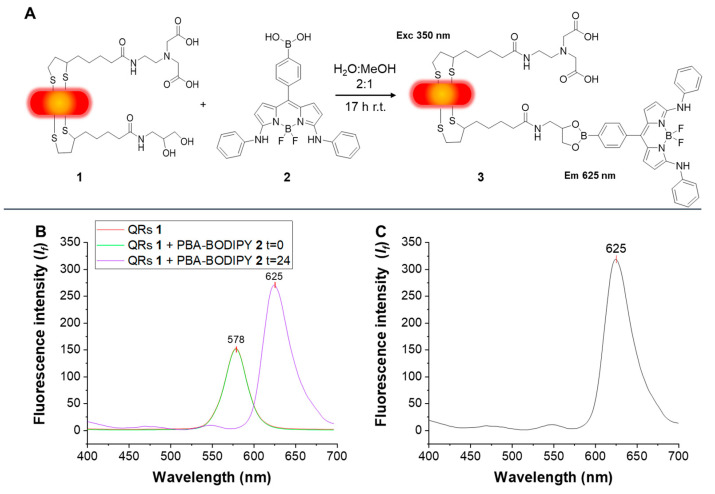
(**A**) Synthesis QRs **3**; (**B**) fluorescence emission spectra (0.05 mg/mL in water) upon excitation at λ_exc_ = 350 nm of a dispersion of QRs **1** (red) and the reaction mixture QRs **1** and PBA-BODIPY **2** at t = 0 (green) and at t = 24 h (cyan); (**C**) QRs **3** (0.05 mg/mL in water) fluorescence emission spectrum upon excitation at λ_exc_ = 350 nm.

Then, PBA-BODIPY **2** was prepared by following a previously reported protocol [20]. The emission bands of QRs **1** (λ_em_ = 578 nm, Figure 3A) specially matched the excitation peak (λ_exc_ = 589 nm) of PBA-BODIPY **2** [20] (Figure 3C), thus suggesting the feasibility of the proposed FRET system. The formation of the five-membered ring on the surface of QRs **1** by the reaction between the 1,2-diol residue on the QRs surface and the PBA residue of the dye **2** was monitored by UV–vis (Figure 4A,B).

The FRET system is thus based, in our hypothesis, on the following steps: (a) excitation of the system at λ = 350 nm; (b) absorption of the radiation by QRs and emission in fluorescence with a peak at λ = 578 nm; (c) energy transfer to the PBA-BODIPY **2** via non-radiative dipole–dipole interactions, which provide an emission peak at λ = 625 nm (Figure 4B,C). Accordingly, QRs **1** were reacted by simply mixing its dispersion in a water/methanol mixture (2:1, see experimental) with the PBA-BODIPY **2** (Figure 4A). Soon after the addition of **2**, the fluorescence peak at λ = 578 nm (green line, Figure 4B) was observed (with λ_exc_ = 350 nm) related to the unconjugated QRs **1**. After 24 h, the fluorescence peak at λ = 625 nm (violet line, Figure 4B), related to the conjugated PBA-BODIPY **2**, was recorded, while the peak at λ = 578 nm disappeared, as expected, due to QRs depletion. Then, the nanoparticles were collected by centrifugation and washed with methanol to remove the residual unreacted PBA-BODIPY **2**. The solid was then dispersed in methanol (0.05 mg/mL), and upon excitation at λ = 350 nm, the spectrum showed an emission peak at λ = 625 nm (black line, Figure 4C), hence confirming the FRET effect of the nanosystem and its stability after purification. Further, DLS analysis of an aqueous dispersion of **3** showed an increase in the NPs’ dimension, from 38 nm in QRs **1** to 72 nm in QRs **3** (see Supplementary Materials, Figure S5), as a result of the conjugation with the PBA-BODIPY **2**. ^1^H-NMR (see Supplementary Materials, Figure S6) of a QRs dispersion in CD_3_OD confirmed the presence of the PBA-BODIPY 2 [20].

**Figure 5:**
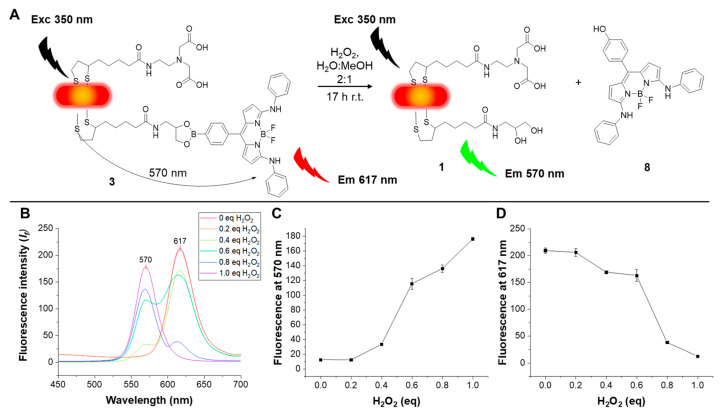
(**A**) Schematic representation of titration of QRs **3** with hydrogen peroxide; (**B**) emission spectra of the reaction mixture after excitation at 350 nm and addition of H_2_O_2_ aliquots; (**C**,**D**) QRs **1** emission intensity at 570 nm and QRs **3** emission at 617 nm upon addition of H_2_O_2_ upon excitation at 350 nm.

To further prove the formation of the boronate esters, a titration with hydrogen peroxide was performed (Figure 5). It is well known that boronic esters are sensitive to the oxidative environment due to the rapid cleavage of the B-O bond under oxidative conditions [33]. In particular, the B-O bond rapidly reacts with H_2_O_2_, and the reaction occurs through oxidation of the B-C bond; the hydrogen peroxide coordinates the boron atom, and then the aryl group migrates to give the boronate intermediate that rapidly hydrolyzes to form phenol and boronic ester [34,35]. Therefore, a titration experiment with QRs conjugate 3 and H_2_O_2_ was performed to confirm the reversibility of this turn-off FRET system (Figure 5A).

A water dispersion of the QRs conjugate **3** was titrated with a solution of hydrogen peroxide, and the reaction was monitored by measuring the emission band upon excitation at λ_exc_ = 350 nm (Figure 5C,D). The H_2_O_2_ solution was added to QRs **3** dispersion in aliquots of 0.2 equivalent (eq), compared to the estimated amount of DHLA-diol **5** in QRs **1**. Looking at fluorescence at λ_em_ 617 nm, a significant variation can be observed in H_2_O_2_ addition (Figure 5D). Simultaneously, the emission band of QRs **1** at 570 nm (Figure 5C) is progressively restored as H_2_O_2_ is added to the mixture. The titration experiment demonstrated that QRs **1** are capable of working efficiently as a turn-off FRET system and are able to detect reactive oxygen species, as proved with hydrogen peroxide.

## 4. Conclusions

Significant efforts have been reported so far for the identification of straightforward QD surface coating strategies for the preparation of efficient QD–organic dyes for FRET-based biosensors. In this work, the conjugation of aniline-disubstituted PBA-BODIPY **2** with bright emission and high quantum yield to water-soluble CdSe ZnS-coated QRs was exploited using the chemistry of phenylboronic acid and diols. The overlapping QRs emission and PBA-BODIPY absorption resulted in a Förster resonance energy transfer effect. This reactivity not only provides a simple and highly specific method for QR functionalization but also inserts a boronic ester bridge that is sensitive to hydrogen peroxide, providing sensing ability to the system.

## Data Availability

Data are available upon request to the corresponding authors.

## Supplementary Materials

The following supporting information can be downloaded at: https://www.mdpi.com/article/10.3390/nano14090794/s1. Figure S1: Representative STEM micrograph overview at low mag of CdSe/ZnS QRs 7; Figure S2: Raw autocorrelation function for the DLS analysis of QRs **1**; Figure S3: ^1^H-NMR (400 MHz) of a dispersion of QRs **1** in D_2_O; Figure S4: ^1^H-NMR spectra comparison: in blue, ^1^H-NMR 400 MHz of DHLA-EDADA **4** in D_2_O; in green, ^1^H-NMR 400 MHz of QRs **1** in D_2_O; and in red, ^1^H-NMR 400 MHz of DHLA-diol **5** in CD_3_OD; Figure S5: Size distribution measured by dynamic light scattering of a dispersion of QRs **3** in water; Figure S6: ^1^H-NMR (400 MHz) of a dispersion of QRs **3** in CD_3_OD; Figure S7: ^1^H-NMR (400 MHz) of DHLA-diol **5** in CD_3_OD; Figure S8: gCOSY NMR (400 MHz) of DHLA-diol **5** in CD_3_OD; Figure S9: gHSQC NMR (400 MHz) of DHLA-diol **5** in CD_3_OD; Figure S10: ^13^C-NMR (100 MHz) of DHLA-diol **5** in CD_3_OD.
