# Supplementary material for: CdSe/ZnS Quantum Rods (QRs) and Phenyl Boronic Acid BODIPY as Efficient Förster Resonance Energy Transfer (FRET) Donor–Acceptor Pair
Source: Nanomaterials (Basel). 2024 May 3;14(9):794. doi: 10.3390/nano14090794 (PMC11085751; doi:10.3390/nano14090794)
Supplement: Supplementary file 1 [file nanomaterials-14-00794-s001.zip › nanomaterials-2915900-supplementary.pdf]

Example of numeration

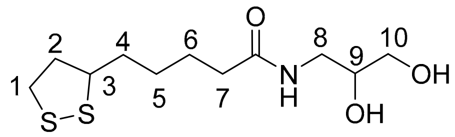

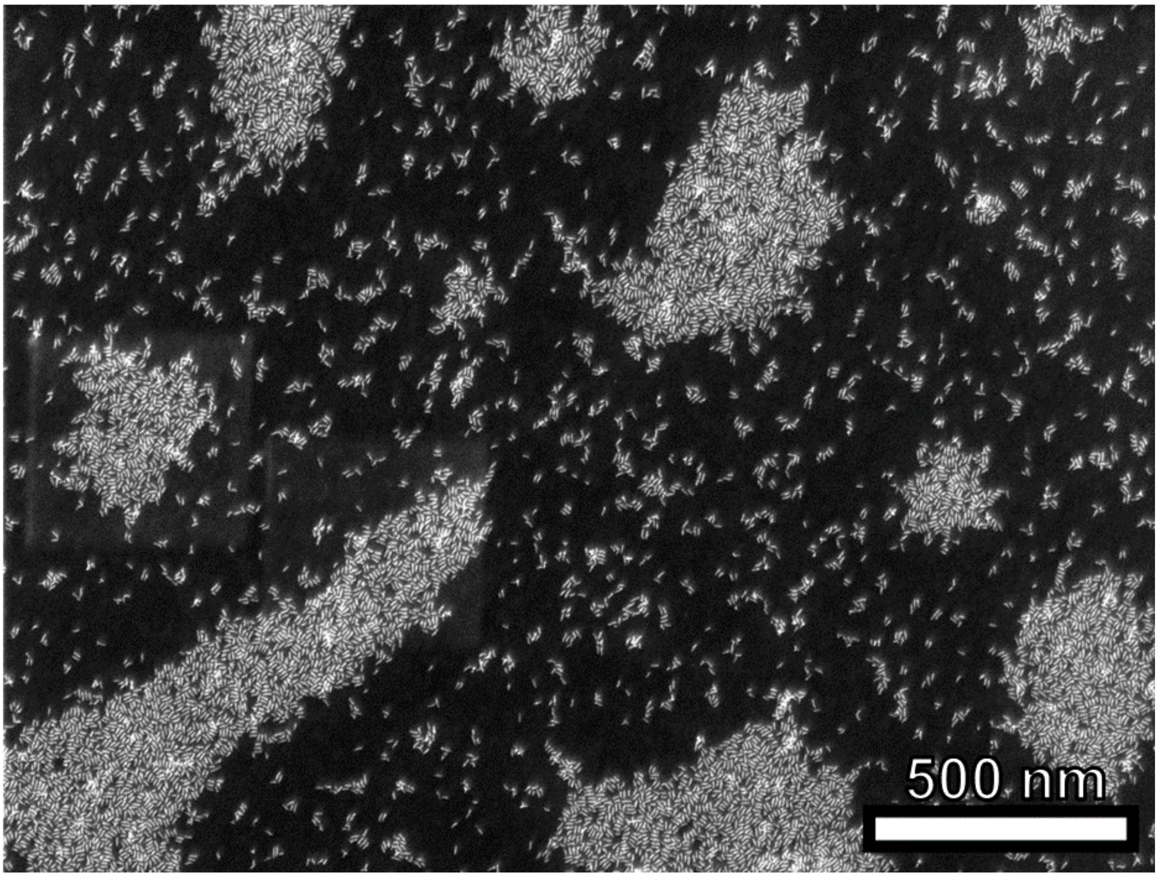

Figure S1. Representative STEM micrograph overview at low mag of CdSe/ZnS QRs **7**.

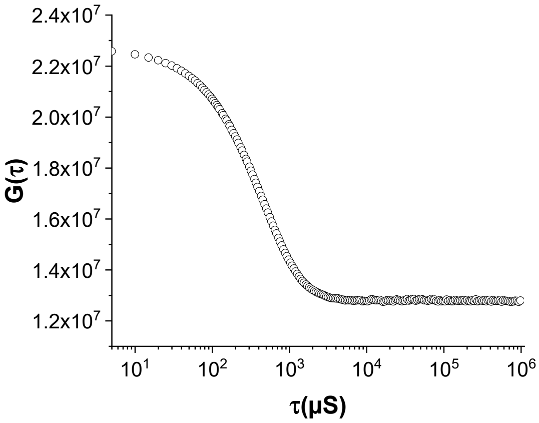

Figure S2. Raw autocorrelation Function for the DLS analysis of QRs **1**.

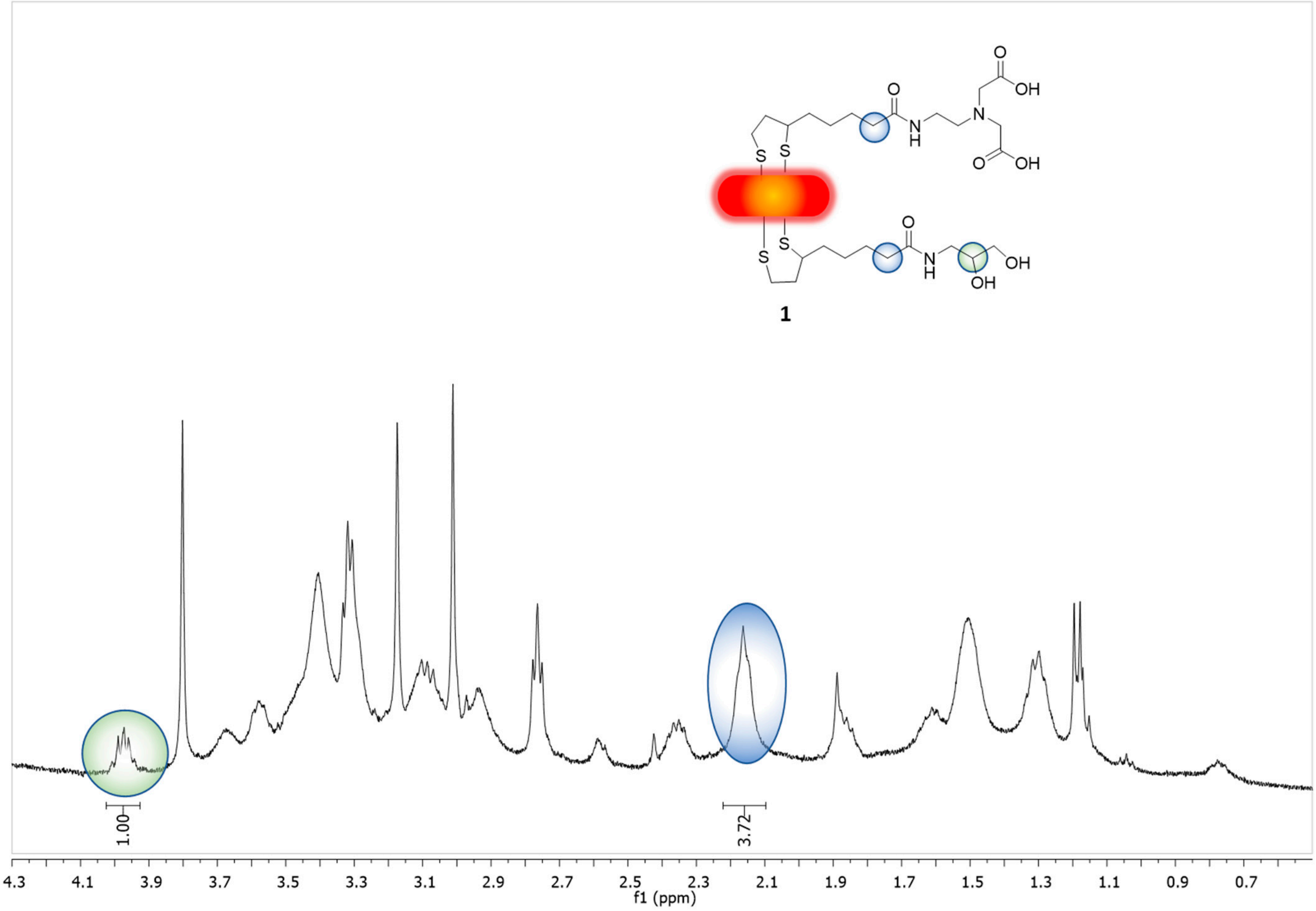

Figure S3. ^{1}H-NMR (400 MHz) of a dispersion of QRs **1** in D_2O.

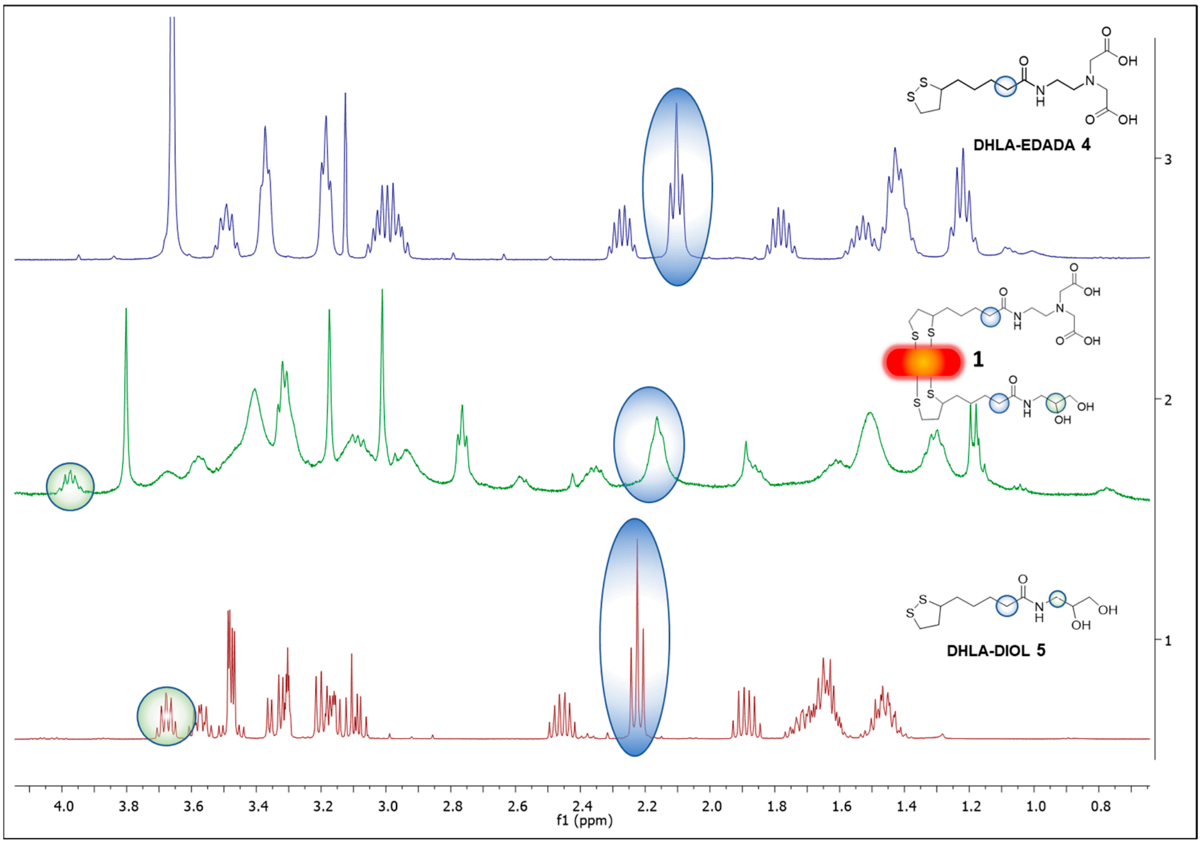

Figure S4. ^{1}H-NMR spectra comparison: in blue ^{1}H-NMR 400 MHz of DHLA-EDADA **4** in D_2O; in green ^{1}H-NMR 400 MHz of QRs **1** in D_2O and in red ^{1}H-NMR 400 MHz of DHLA-DIOL **5** in CD_3OD.

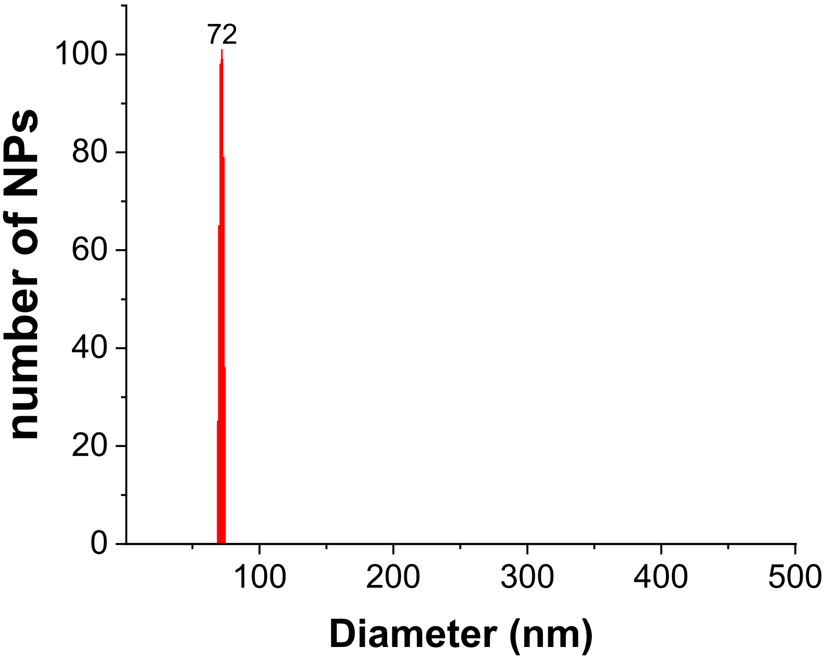

Figure S5. Size distribution measured by dynamic light scattering of a dispersion of QRs **3** in water.

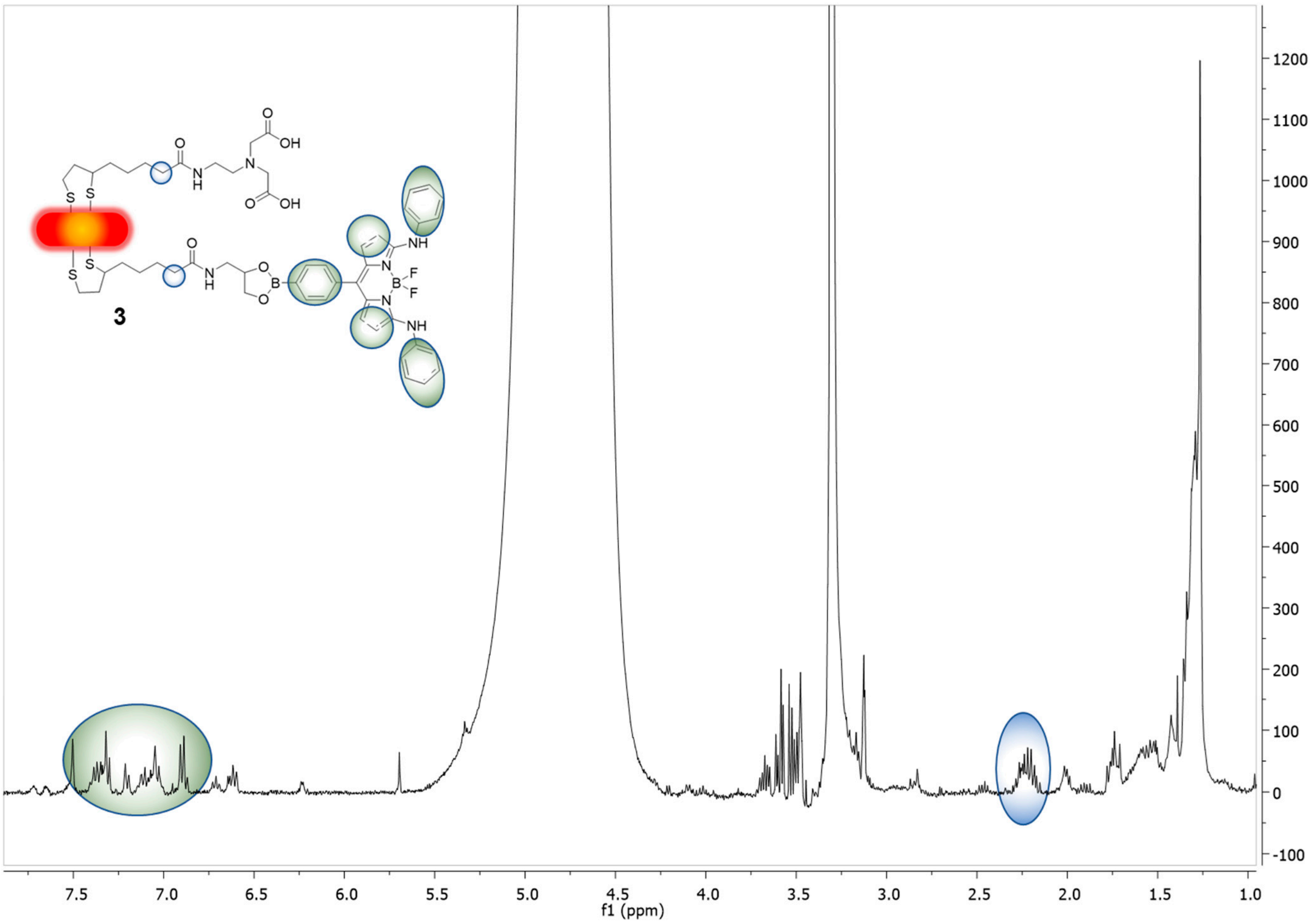

Figure S6. ^{1}H-NMR (400 MHz) of a dispersion of QRs **3** in CD_3OD.

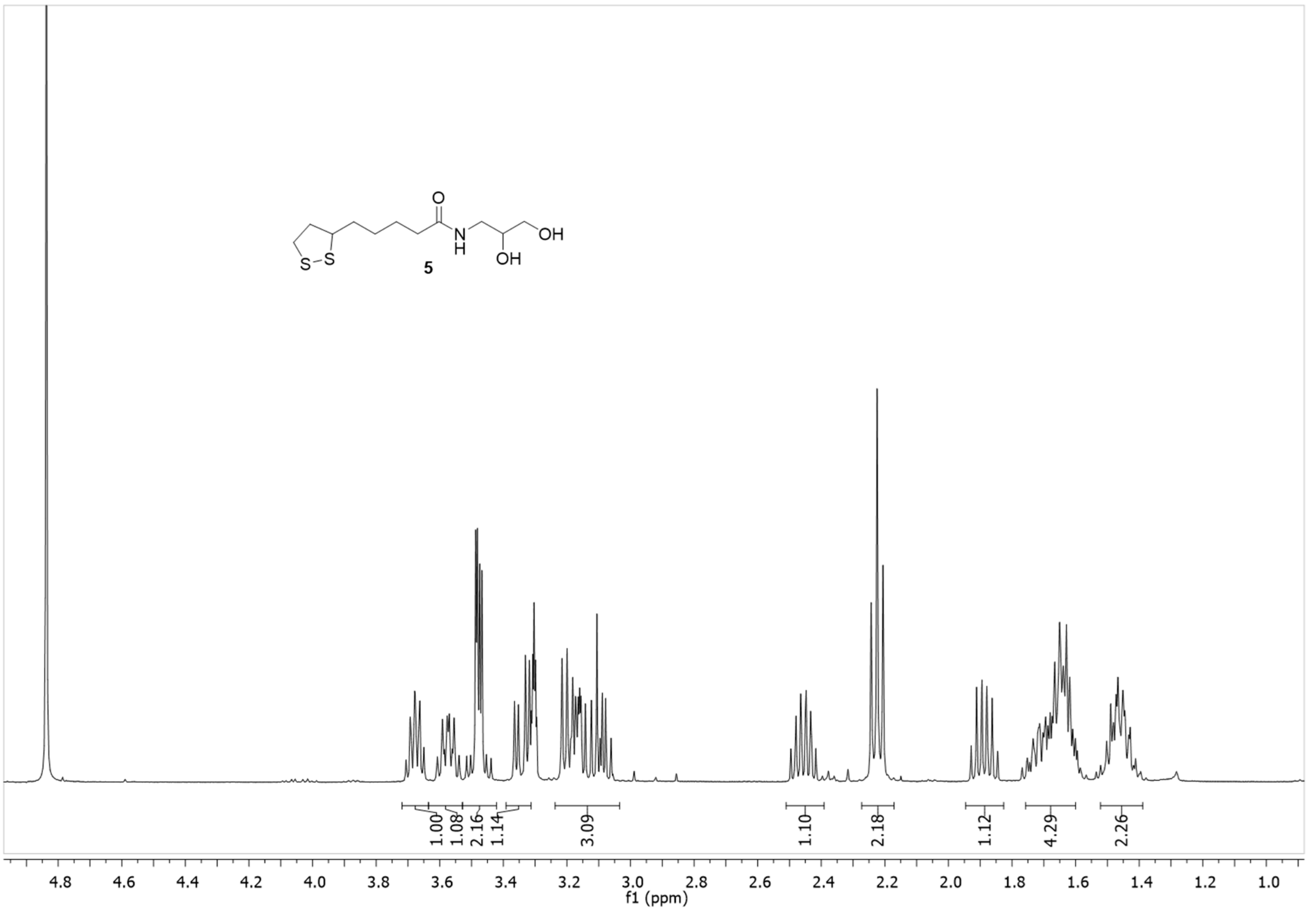

Figure S7. ^{1}H-NMR (400 MHz) of the DHLA-Diol **5** in CD_3OD.

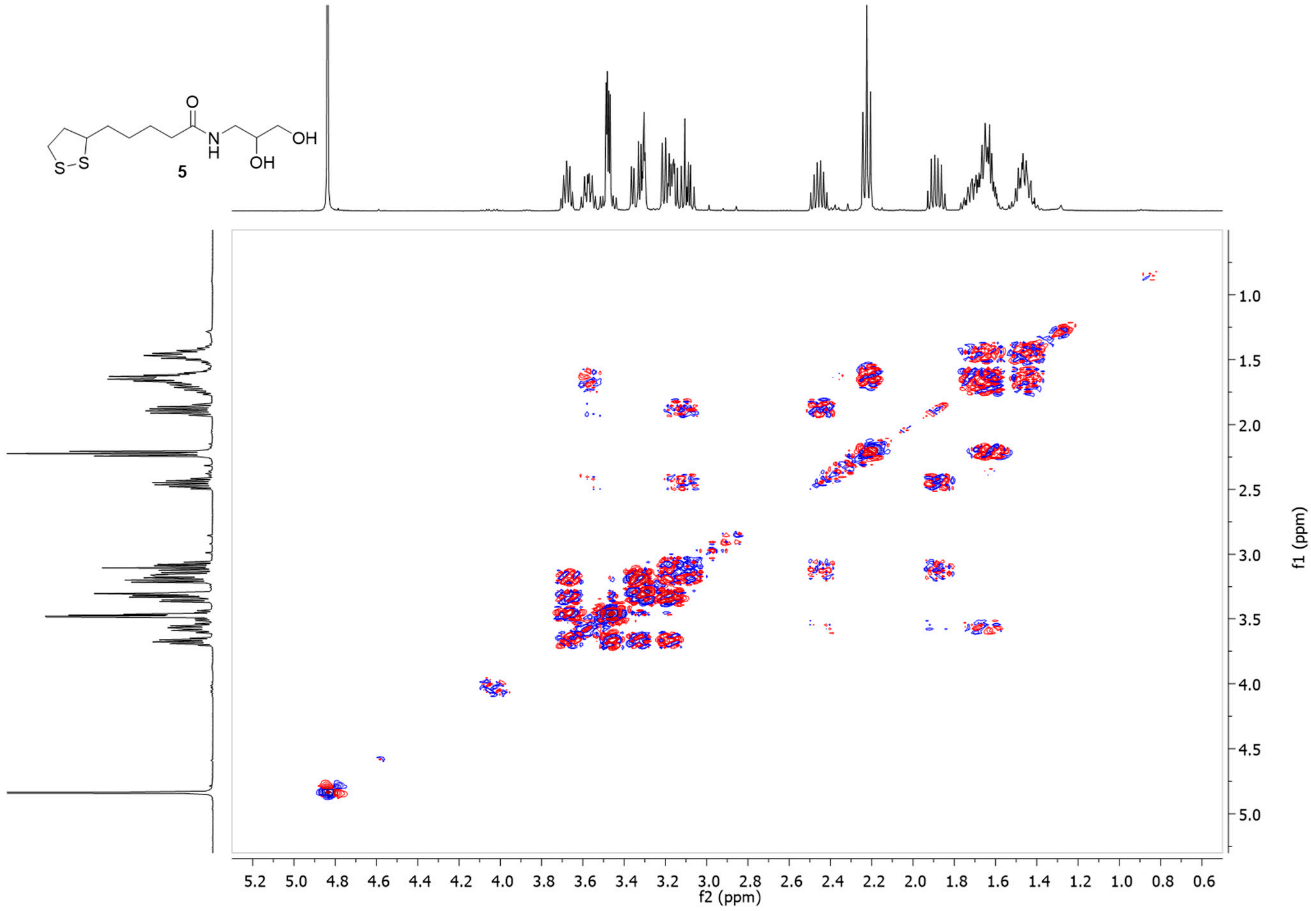

Figure S8. gCOSY NMR (400 MHz) of the DHLA-Diol **5** in CD_3OD.

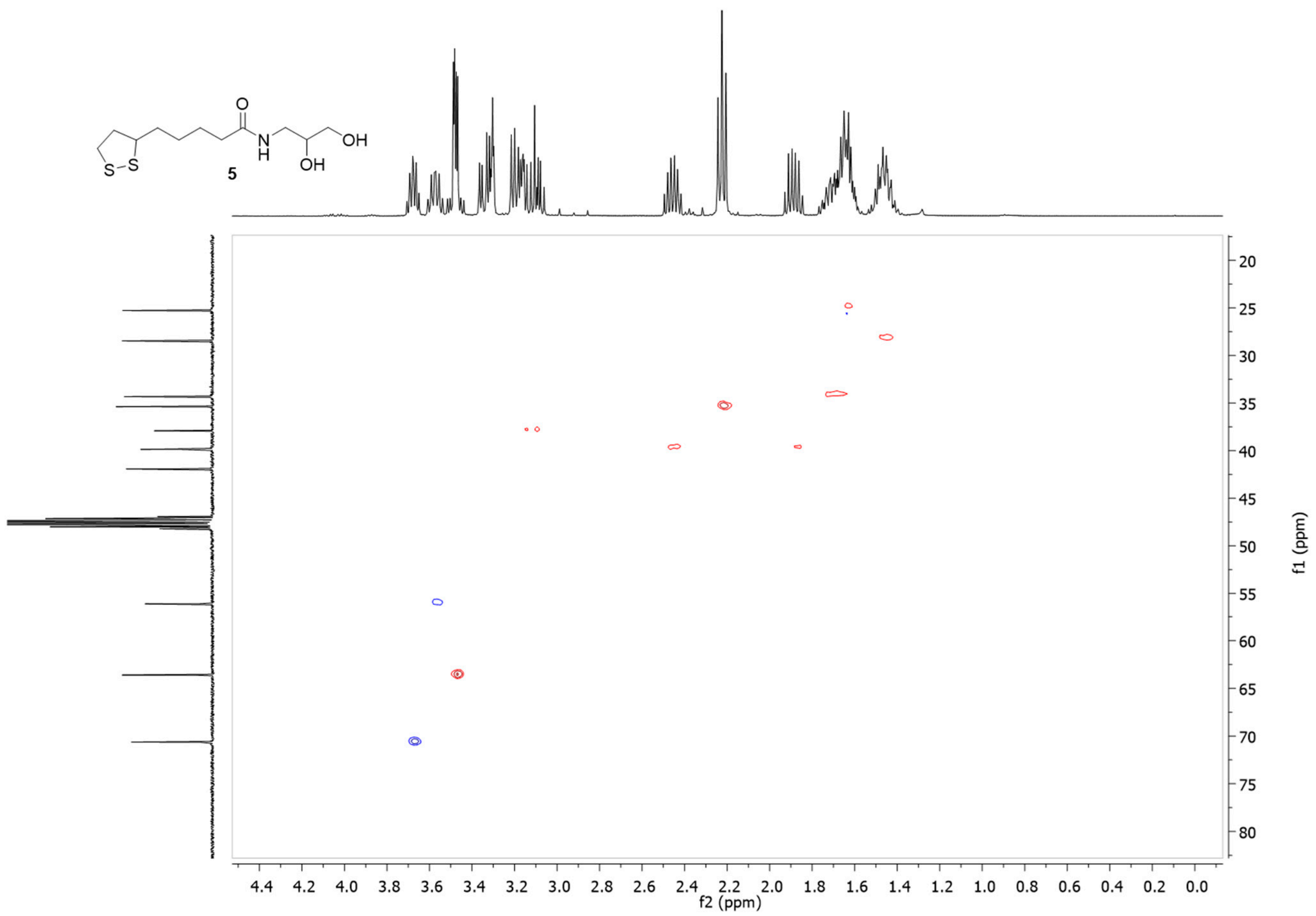

Figure S9. gHSQC NMR (400 MHz) of the DHLA-Diol **5** in CD_3OD.

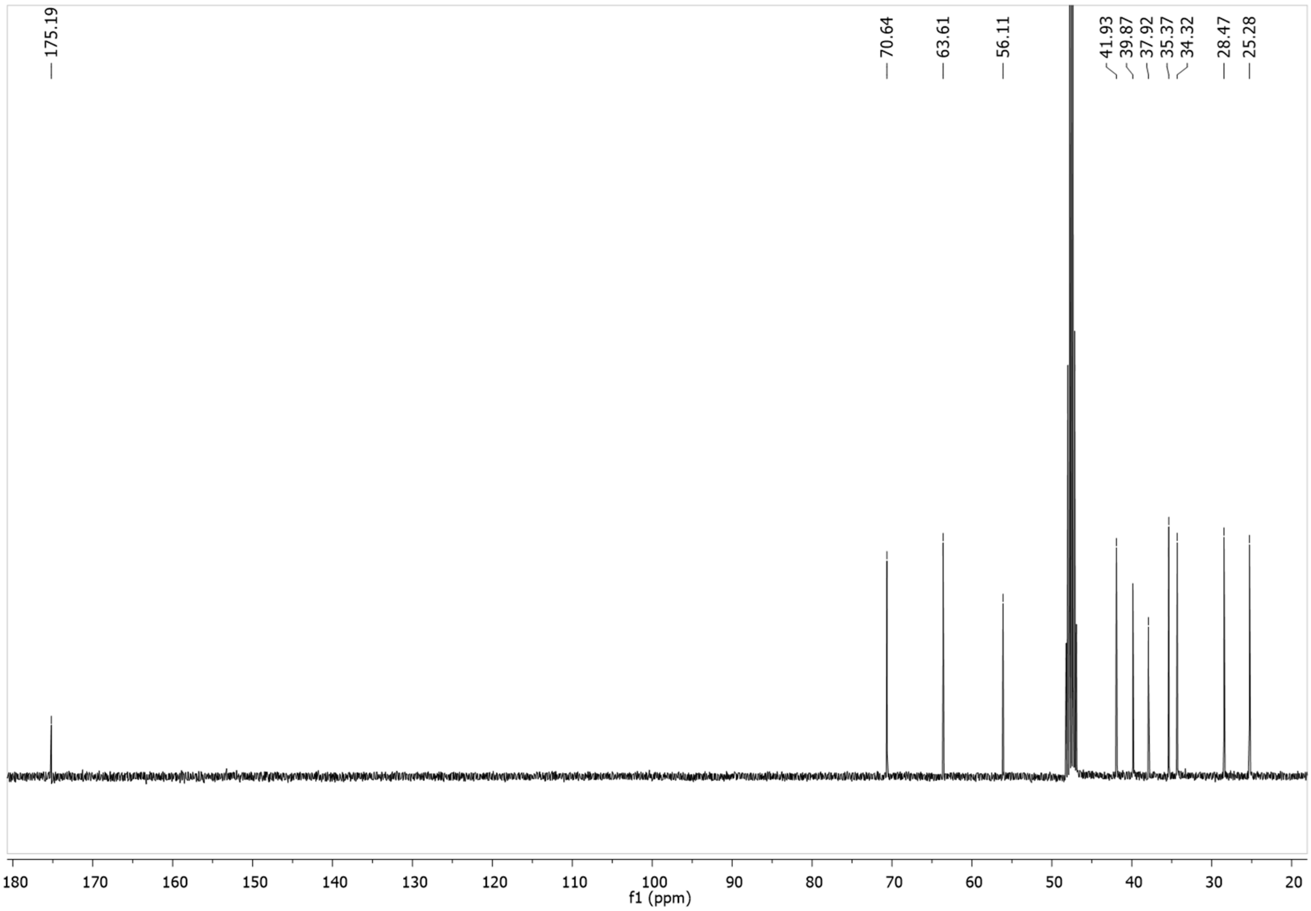

Figure S10. ^{13}C-NMR (100 MHz) of the DHLA-Diol **5** in CD_3OD.
